# Impact of immunotherapy as a monotherapy or in combination with conventional therapies on regulatory T-cell (Treg) modulation in prostate cancer: a systematic review

**DOI:** 10.1186/s12885-026-16550-w

**Published:** 2026-07-17

**Authors:** Olayemi Oluseun Akinnola, Ayomide Iyanuoluwa Olaniyan, Barnabas Adeoluwa Adejoye, Abimbola David Akinyosoye

**Affiliations:** 1 Department of Biological Sciences, Covenant University, Ota, Ogun State Nigeria; 2 Covenant Applied Informatics and Communication Africa Centre of Excellence (CApIC-ACE), Covenant University, Ota, Ogun State Nigeria

**Keywords:** Combination Therapy, Immunotherapy, Immune Priming, Monotherapy, Prostate Cancer, Regulatory T Cells, Tumor Microenvironment

## Abstract

**Background:**

Prostate cancer is known to have a cold tumor microenvironment (TME) that promotes immune evasion through different cellular pathways. Aggressive disease progression is often linked to Regulatory T cells (Tregs) which play an important role in immunosuppression. While immunotherapy aims to use the immune system to fight tumor cells, its efficacy is frequently hindered by Tregs, which prevent effector T cells from reaching and fighting tumor cells. This review evaluates the impact of different immunotherapeutic interventions, alone or in combination, on Treg frequency and function within the prostate TME.

**Methods:**

Following PRISMA guidelines, a systematic search of major databases identified studies investigating immunotherapeutic effects on Tregs in prostate cancer. Studies were included if they evaluated monotherapies or combination strategies, as defined by inclusion criteria.

**Results:**

31 studies met the inclusion criteria. Qualitative analysis revealed that monotherapies, including immune checkpoint inhibitors and vaccines, produced inconsistent effects on Treg populations, which in many cases did not significantly decrease their levels or suppressive function. Notable exceptions included targeted recruitment inhibitors such as anti-CCR4 and specific viral-mediated therapies. In contrast, combination therapies proved to be more effective in reshaping the TME. When immunotherapy and other treatment such as androgen deprivation therapy, immunogenic chemotherapy, and targeted molecular inhibition are combined, enhanced anti-tumor immunity is observed.

**Conclusion:**

Successful modulation of the prostate TME requires shifting from monotherapy toward combination strategies that overcome Treg-mediated immunosuppression. Effector-to-Treg ratios and functional markers, rather than Treg frequency alone, may better predict outcomes, as CD8⁺ infiltration does not always reflect restored cytotoxicity. These findings highlight the dual potential of Tregs as therapeutic targets and biomarkers in prostate cancer.

**Supplementary Information:**

The online version contains supplementary material available at https://doi.org/10.1186/s12885-026-16550-w.

## Background

Prostate cancer remains one of the major causes of death amongst men across continents of the world, ranking second most frequently diagnosed malignancy in men contributing to the overall cancer mortality with the aging population having a greater increasing burden [1]. Radical prostatectomy remains a cornerstone treatment for localized prostate cancer and provides durable cancer control in appropriately selected patients [2]. For advanced disease, androgen deprivation therapy (ADT) is the mainstay of treatment and is usually effective initially; however, many patients eventually develop castration-resistant prostate cancer (CRPC) [3] and metastatic castration-resistant prostate cancer (mCRPC) [4]. Once this transition occurs, therapeutic options become substantially more limited. mCRPC is associated with high morbidity and poor survival outcomes and is characterized by resistance to castration-level testosterone and often displays biological and genomic complexity that limits therapeutic durability [4]. These limitations highlight the need for therapeutic strategies to beat tumor adaptation and resistance mechanisms.

The prostate TME helps support immune evasion through different cellular and molecular pathways that suppress antitumor immunity. Key immunosuppressive populations include Tregs, myeloid-derived suppressor cells (MDSCs), tumor associated macrophages (TAMs), and cancer-associated fibroblasts (CAFs) all of which support tumor progression and therapeutic resistance through cytokine- and growth factor-mediated signaling pathways [5, 6]. Recent single-cell transcriptomic studies have further delineated the cellular heterogeneity of the prostate TME, identifying distinct immune and stromal subpopulations including FOXP3⁺ Tregs, exhausted CD8⁺ T cells, and CXCL14⁺ CAFs whose composition changes substantially during disease progression and the development of castration resistance [7, 8]. Among these populations, M2-type TAMs, predominate within the TME, promote castration resistance and neuroendocrine differentiation through HMGB1-induced IL-6 signaling. MDSCs contribute to therapeutic resistance by suppressing T-cell and NK-cell activity, promoting Treg expansion, and activating AR downstream signaling through IL-23 secretion. CAFs also engage in bidirectional communication with prostate cancer cells and drive resistance to anti-androgen therapy and chemotherapy through NRG1-, SPP1-, and TGF-β-mediated pathways [6]. Together, these populations create an immunosuppressive microenvironment that limits treatment efficacy and facilitates tumor progression.

Immunotherapy includes a wide range of treatments that harness the patient’s own immune system to target and eliminate tumor cells, ranging from passive strategies involving external agents such as exogenous cytokines or monoclonal antibodies, to active strategies that boost the body’s natural anti-tumor response [9]. Common immunotherapy approaches include immune checkpoint inhibitors, therapeutic cancer vaccines, adoptive T-cell therapies, cytokine-based therapies, and bispecific antibodies or immune modulators [9–11].

Research has shown that single-agent immunotherapy’s efficacy is hindered by the tumor’s ability to evade the immune system marked by low mutational burden, sparse tumor-infiltrating lymphocytes, T-cell exclusion, and an immunosuppressive microenvironment driven by Treg cells, MDSCs and TAMs [11]. Response rates in unselected patients remain extremely low despite the promise of monotherapy, with overall survival benefits among approved agents and pembrolizumab restricted to MSI-high/dMMR or high-TMB tumors [10]. The prevalence of MMR mutations varies across ethnic populations, with pathogenic MMR mutations reported more frequently in metastatic Chinese prostate cancer patients than in Caucasian cohorts (4.8% vs. 2.2%, *p* = 0.006), highlighting the need to consider population-specific differences when selecting patients for ICB [12]. In addition, intratumoral CD8 + T-cell density has emerged as a promising predictor of response in dMMR/MSI-H patients [12]. Recent single-cell profiling studies have further revealed molecular subtypes with distinct immune landscapes, with immune-enriched subtypes showing elevated LAG3 expression and greater predicted responsiveness to immunotherapy [7]. Together, these findings emphasize the importance of biomarker-guided patient selection and suggest that durable responses will likely require strategies capable of reprogramming the immunosuppressive tumor microenvironment through rational combinations that enhance antigen release, T-cell priming, trafficking, and persistence [9, 10].

Tregs are one of the widely known subsets of immunosuppressive cells that plays a crucial role in maintaining immune homeostasis, suppressing overreaction and immunity actions against healthy cells (self-antigens). To do this, Tregs suppress autoreactive effector T cells and control inflammation, employ diverse mechanisms that include secretion of immunosuppressive cytokines (IL-10, TGF-β, IL-35), CTLA-4-mediated inhibition of antigen-presenting cell maturation, metabolic disruption via CD39/CD73-generated adenosine, granzyme/perforin-dependent cytolysis, and release of immunoregulatory extracellular vesicles. However, cancer cells hijack this mechanism to evade immune surveillance and destruction by immune cells thereby creating an immunosuppressive environment that facilitates tumor progression. These Tregs are a specialized subset of CD4⁺ T lymphocytes which consist of high amount of a protein called interleukin-2 receptor α-chain (CD25) on their surface responsible for Treg’s growth and function, and a special transcription factor forkhead box P3 (FOXP3^+^) that acts like a master switch that helps Tregs develop and function. Tregs consist of natural and induced subsets, with further functional divisions into resting, effector, memory like and activated subsets, all characterized by markers CTLA-4, TIGIT, CCR8 and other molecules [13].

Since Tregs are strong inhibitors of CD8⁺ cytotoxic T cells and NK cells via contact-dependent and cytokine-mediated pathways, allowing tumor immune escape and treatment resistance, elevated Treg levels in Pca are frequently observed in patients with advanced disease stages, aggressive disease progression, bone metastases, and poor survival outcomes. Tregs are drawn into the prostate TME by chemokines, including CCL22, CCL20, and CXCL12 that binds CCR4/CXCR4 receptors on Tregs, whereas in bone marrow metastases, Tregs interact with RANKL and promote Treg proliferation and immune suppression [14, 15]. Thus, Tregs play a key role in maintaining prostate cancer’s immunologically “cold” nature.

Immunotherapy affects the frequency, phenotype, and function of Tregs. As a result, Tregs play a big role in deciding how well cancer treatment works. Immunity can be enhanced by using CTLA-4 blockade that depletes intratumoral FOXP3 + Tregs via antibody-dependent cellular cytotoxicity (ADCC) [16]. PD-1 blockade, however, may increase or activate certain Treg populations, since Tregs expressing PD-1 can acquire more suppressive activity once PD-1 is inhibited [17, 18]. Other treatments such as cancer vaccines and cytokine-directed therapies can also alter Tregs by boosting effector T-cell activation. As a reaction to a higher influx of T cell, the body may produce more Tregs to prevent the immune system from over-reactivity as a feedback mechanism, maintaining immune homeostasis. TME can be reshaped by a combination of treatment modalities such as ICI plus radiotherapy, androgen deprivation, or chemotherapeutic agents, altering how Tregs move into the tumor, how long they survive, and how strongly they suppress immune responses [11].

Changes in Treg levels during immunotherapy have significant clinical implications, influencing treatment response, progression-free survival (PFS), and overall survival (OS). Elevated intratumoral Tregs are broadly associated with immune suppression and poorer prognosis, whereas decreasing Tregs, particularly FOXP3⁺ effector Tregs have been linked to better clinical results in ICI-treated patients [16, 19]. Evidence has shown that blocking CTLA-4 can reduce Tregs, which is associated with better anti-tumor outcomes and stronger CD8⁺ effector T-cell activity [20]. On the other hand, the increase in suppressive Treg populations during PD-1 therapy has been linked to treatment resistance and disease progression, suggesting that Tregs can be used as biomarkers to identify people who are not responding well to therapy. Tregs suppress antitumor immunity through antigen-presenting cells (APC) suppression, IL-2 depletion, and release of immunosuppressive cytokines [21]. Since these pathways have a direct effect on immunotherapy success, monitoring Treg levels can be used as a possible approach to predicting response, guide patients’ stratification, and informing combination therapies. Overall, this highlights the value of Tregs as promising biomarkers in prostate cancer immunotherapy [19].

## Materials and methods

This systematic review used the Preferred Reporting Items for Systematic Reviews and Meta-Analyses (PRISMA) 2020 guidelines [22].

### Inclusion and exclusion criteria

The studies included in this systematic review were selected based on specific criteria to ensure the relevance and quality of the data. Articles published between 2015 and 2025, written in English, and addressing the impact of immunotherapy on Tregs in prostate cancer were included. Eligible studies had to compare immunotherapeutic interventions (monotherapy or combinations) with standard of care, placebo, or no immunotherapy controls across all clinical stages of prostate cancer or in preclinical models. Exclusion criteria included case reports, studies that did not address the Population, Intervention, Comparison, and Outcome (PICO) framework, duplicate studies, or studies lacking original immunological data.

### Search strategy

The search strategy was designed to identify relevant studies in the PubMed, Web of Science, Cochrane Library, and Google Scholar databases. It was based on the PICO tool as follows: (P) population: men diagnosed with localized, advanced, or metastatic prostate cancer, as well as preclinical prostate cancer models; (I) intervention: cancer immunotherapies including checkpoint inhibitors, therapeutic vaccines, adoptive T-cell therapies, and cytokine therapies, used alone or in combination with conventional treatments (androgen deprivation therapy, chemotherapy, or radiotherapy); (C) control: standard of care, placebo, or no-immunotherapy controls; (O) outcomes: changes in Treg frequency, subsets, or immunosuppressive function and impact on treatment response or survival.

### Study selection and data extraction

Study selection was independently conducted by two reviewers through a manual screening process of titles and abstracts. This initial phase aimed to identify potentially relevant studies based on the predefined eligibility criteria, with any discrepancies between the two reviewers resolved through consensus or by consultation with a third reviewer to reach a final decision.

The data extraction was performed by a single reviewer and double-checked by a second reviewer to ensure accuracy. Extracted data were compiled into a dedicated database for subsequent qualitative synthesis.

### Quality assessment

The quality of the included studies was rigorously assessed using standardized appraisal tools tailored to the specific design of each study. Preclinical murine and canine studies were evaluated using the Systematic Review Centre for Laboratory Animal Experimentation (SYRCLE) risk of bias tool [23]. For clinical evidence, randomized controlled trials were assessed using the Cochrane Risk of Bias 2.0 (RoB 2.0) tool [24], while non-randomized studies of interventions were evaluated using the Risk Of Bias In Non-randomized Studies of Interventions (ROBINS-I) framework [25]. This assessment allowed for identifying potential biases and their impact on the results, ensuring the robustness of the review’s conclusions.

**Figure:**
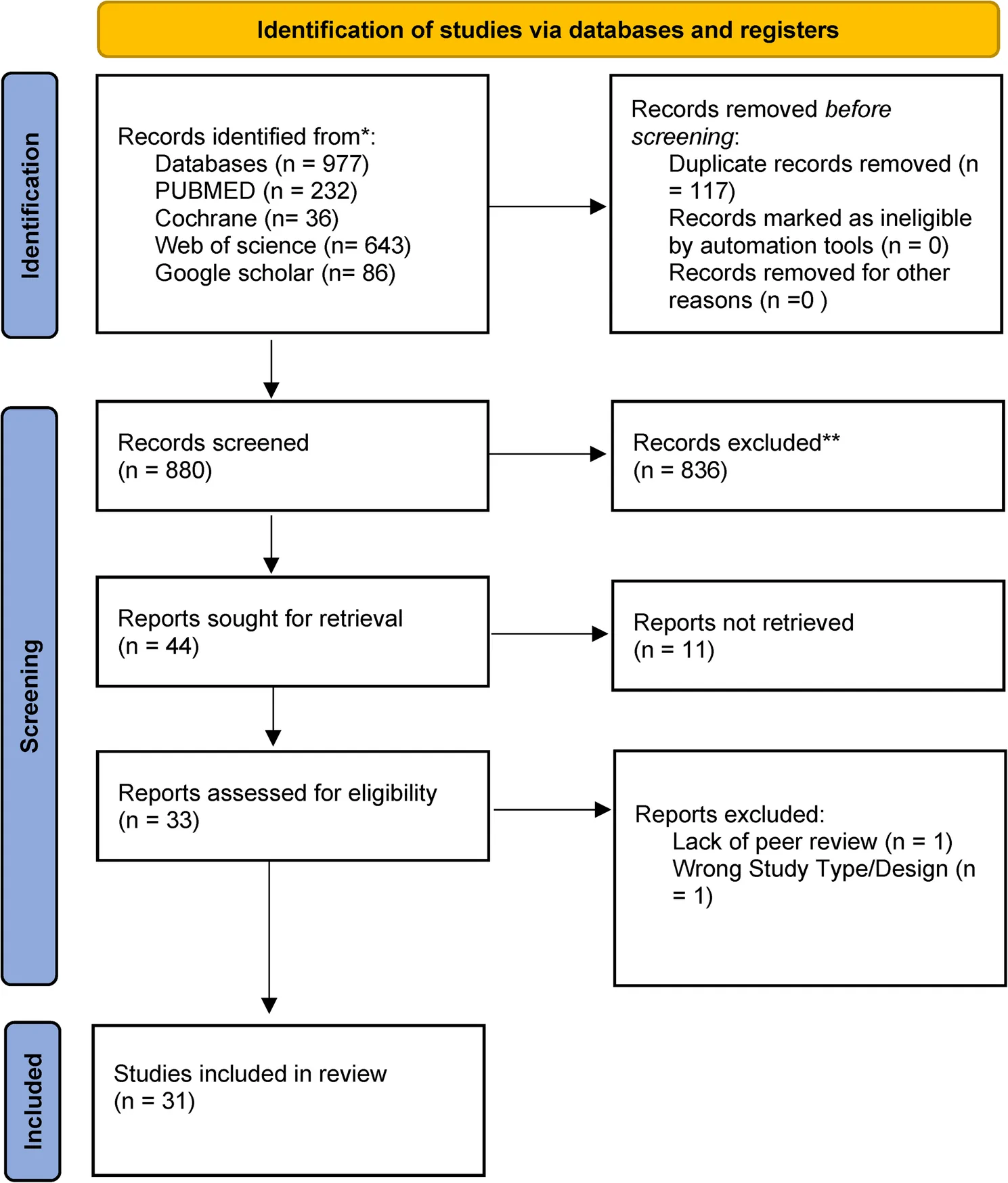

## Results

### Quality assessment of included studies

The quality of randomized clinical studies was evaluated through the RoB 2.0 tool, indicating that most trials presented some concerns and only one study presented a low risk, due to the deviations from intended interventions (Fig. S2). For preclinical studies, the quality was assessed using the SYRCLE tool which showed a predominantly moderate risk of bias. This was largely due to incomplete reporting across multiple domains (Fig. S1). The non-randomized clinical studies were assessed using the ROBINS-I tool, and all were found to be at a serious risk of bias. This high risk was largely attributed to the bias due to confounding domain (Fig. S3). Due to the nature of the procedure being studied, double blinding was not feasible, an inherent limitation in these studies. This introduces potential performance and reporting bias, an inherent limitation of open-label intervention studies. Despite these concerns, the assessment indicates that while there is a risk of bias, the findings from the included studies are still valuable. However, they should be interpreted with caution.

**Fig. 1:**
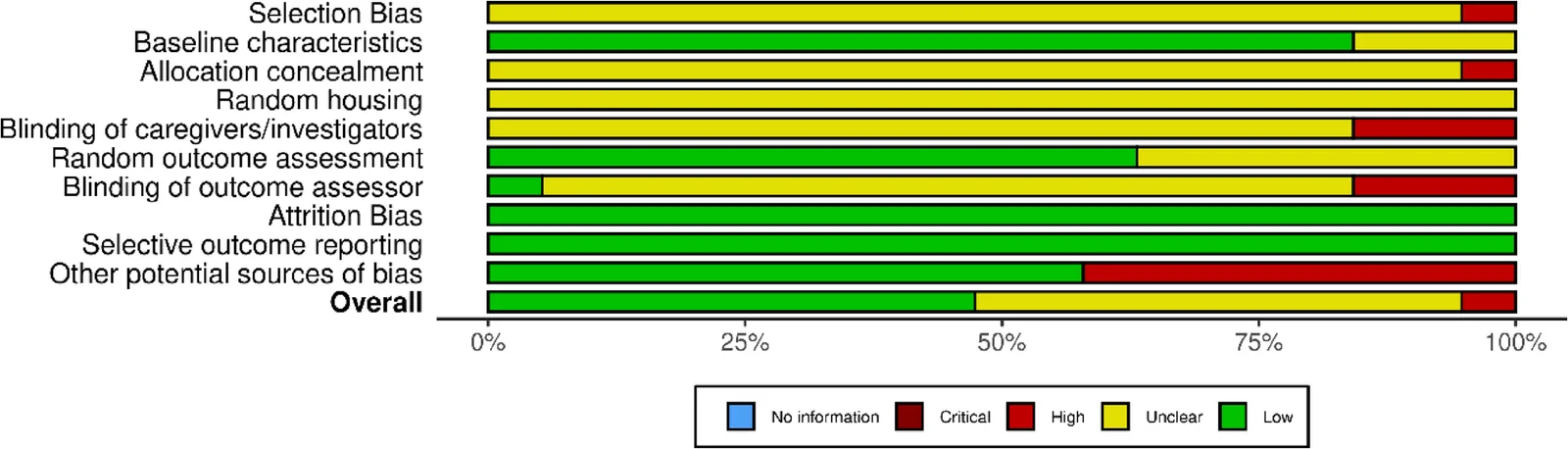
Summary of risk of bias assessment using the SYRCLE tool for preclinical studies

**Fig. 2:**
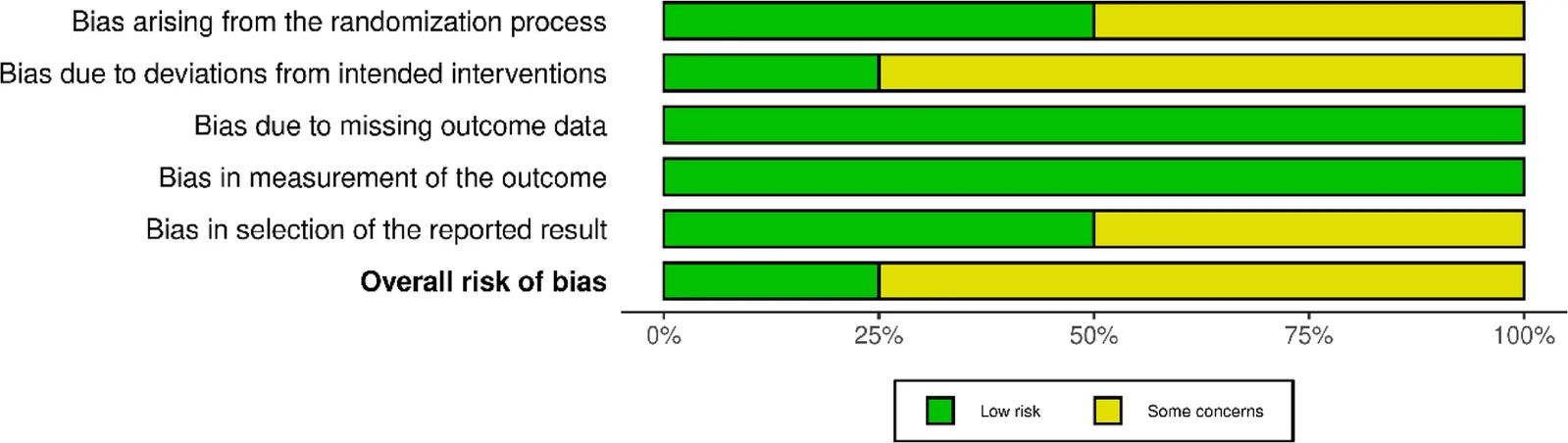
Summary of risk of bias assessment using the ROB 2.0 tool for randomized clinical studies

**Fig. 3:**
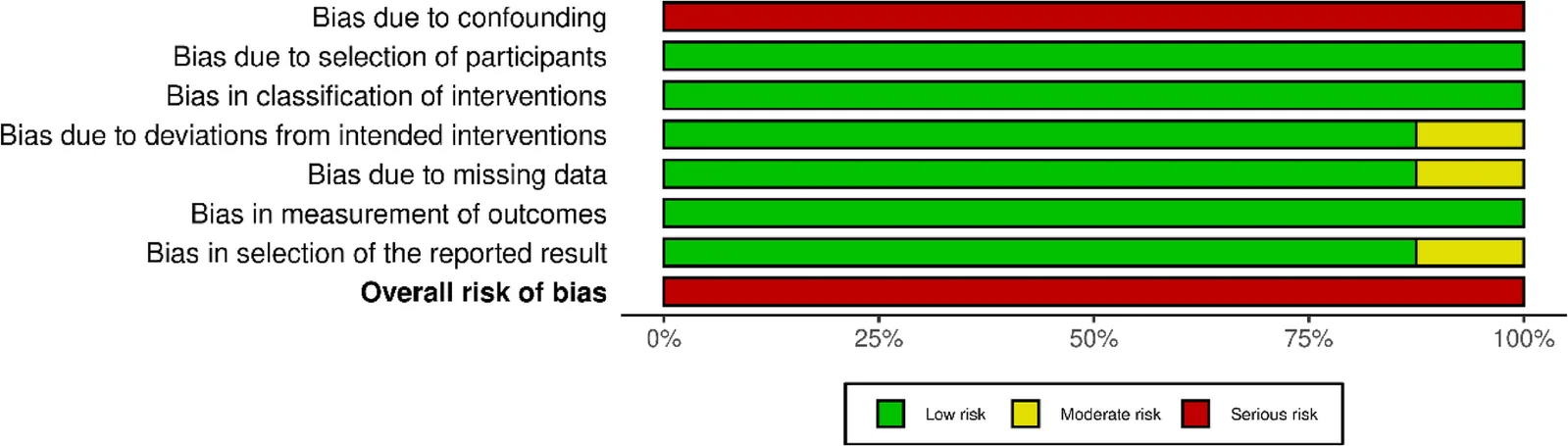
Summary of risk of bias assessment using the ROBINS-I tool for non- randomized clinical studies

The summary of the SYRCLE, RoB 2.0, ROBINS-I assessments and their associated applicability concerns is presented in (Figs. 1, 2 and 3).

### Overview of included immunotherapeutic strategies

In this review, preclinical and clinical trials assessing the impact of immunotherapy on the Tregs in prostate cancer (Table S1) were included. The preclinical studies were mainly done in mouse models such as Myc-CaP, xenografts, and mechanistic in vivo systems, and a canine model for translational relevance. Clinical studies encompassed early-phase trials in localized prostate cancer, metastatic castration-resistant prostate cancer (mCRPC), and high-risk disease, including Phase I, Phase I/II, and translational trials. The comprehensive baseline parameters for all included studies encompassing study designs, specific intervention regimens, designated Treg surface markers, tumor models or clinical stages, sample sizes, and follow-up durations are thoroughly detailed in (Table S4).

Therapies included immune therapies which were either monotherapy or combination therapy. Monotherapies included cancer vaccines, immune checkpoint inhibitors, cytokine-based treatments, and experimental agents targeting immune regulatory pathways. Combination strategies involved use of ADT, chemotherapy, or radiotherapy in combination with immunotherapy, to boost antitumor immunity and eliminate immunosuppression.

Across studies, the results of Tregs were measured by intratumoral and circulating Treg frequency, suppressive activity, FOXP3^+^ expression, and T cell proportions of effector cells to Tregs. Some studies went further to measure adaptive or functional Treg resistance as opposed to abundance. Most preclinical studies reported that Treg depletion or modulation enhanced antitumor immunity whereas some combination therapies were limited by Treg expansion. Clinical studies demonstrated that immunotherapy can enhance effector T-cell infiltration, cause adaptive Treg resistance, or safely modulate immunity, with early signals of therapeutic benefit in mCRPC.

The quality of methodology of the included studies was moderate-to-high in preclinical studies and low-risk to some concerns or serious risk of bias in clinical studies, which also indicates the necessity to interpret the results in the context of study limitations. Considering all these studies, they highlight the diversity of immunotherapeutic interventions in prostate cancer and their impact on immune regulation by Tregs.

### Effects of monotherapies on Treg modulation

Across monotherapy studies (Table S2), immunotherapy had mixed effects on Tregs in prostate cancer showing variable results, with study outcomes ranging from Treg reduction to no change or even Treg accumulation. Several interventions were linked with reduced levels or function of Treg. These included PTEN mRNA nanoparticles [26], CP1 bacterial isolate [27], anti-CCR4 antibody [28], fusion-protein vaccination [29], FLII restoration [30], and HVJ-E viral particles [31], all of which reduced Treg frequency or infiltration and were generally accompanied by improved CD8⁺ T-cell activity or preservation of cytotoxic CD8⁺ populations. For instance, Maeda et al. [28], reported that mogamulizumab selectively depleted circulating CD4⁺FOXP3⁺ Tregs and CCR4⁺ Tregs while leaving CD8⁺ cytotoxic T cells and CD4⁺ helper T cells unaffected, resulting in a more favorable CD8⁺/Treg balance or reversal of immunosuppressive features of the tumor microenvironment. In addition, intervention such as αvβ8-targeting strategies [32] and chromogranin A–derived peptides [33] inhibit Tregs activity by blocking TGF-β pathways while Treg counts remains unaltered. In contrast, several monotherapies showed no significant effect on Treg levels. Personalized peptide vaccination alone [34, 35] and anti-CTLA-4 monotherapy [36] produced limited immune modulation and did not meaningfully alter circulating or intratumoral Tregs in either clinical or preclinical models. Only few studies observed increases or persistence of Tregs, for example, B7-H3 inhibition [37] and systemic GM-CSF [38] led to Treg accumulation or expansion. In vaccine trials [39, 40], elevated baseline or antigen-specific Treg levels were linked to weaker immune responses.

Altogether, Tregs were inconsistently modulated by monotherapies, with some existing approaches having partial or limited effects, supporting ongoing evaluation of combination therapies.

### Effects of combination therapies on Treg modulation

In contrast to monotherapy studies, combination immunotherapies (Table S3) generated more consistent and effective modulation of Tregs compared to monotherapies, particularly within the TME, and were in many cases linked to increased antitumor immune activity. Across preclinical and clinical studies, immunotherapy was shown to be more effective in combination with conventional or immune-priming modalities in enhancing the ability to overcome Treg-mediated immunosuppression.

Preclinical studies showed that combining ADT with intratumoral Treg depletion [41], or checkpoint blockade with immunogenic chemotherapy [42], significantly reduced Treg numbers and improved CD8⁺ T-cell–to-Treg ratios. Importantly several studies went beyond reporting CD8⁺ T-cell abundance and evaluated functional characteristics of these cells. Shen et al. [41] demonstrated that the increase in CD8⁺ T cells following castration was accompanied by functional activation. During the peri-castration period, these cells produced granzyme B, IFN-γ, TNF-α, and IL-2, indicating active cytotoxic and effector functions. The authors also assessed the exhaustion markers PD-1, CTLA-4, LAG-3, and TIM-3 on tumor-infiltrating CD8⁺ T cells and found that their expression increased shortly after castration before declining as castration resistance developed. Similarly, Ager et al. [43] used SPAS-1 tetramer staining to identify tumor-specific CD8⁺ T cells and demonstrated increased TCR repertoire diversity following combination therapy. Other approaches targeting oncogenic or immunosuppressive pathways [34, 44, 45] similarly reduced Treg and other suppressive cell populations while enhancing infiltration of effector T cells. Intrinsic immune activation such as agonists of STING pathway [43] was also shown to remodel the TME to a more immunostimulatory state.

Clinical and translational studies showed similar patterns. Combining dendritic cell–based vaccines with docetaxel [46] or metronomic cyclophosphamide [34] consistently reduced circulating Tregs, promoting systemic immune activation and stronger effector T cell responses. Podrazil et al. [46] reported a significant increase in PSA-specific IFN-γ-producing CD8⁺ T cells following chemoimmunotherapy. Similarly, van Dodewaard et al. [47] found that higher frequencies of ICOS-expressing CD8⁺ T cells, rather than total CD8⁺ T-cell counts, were associated with improved progression-free survival following mitoxantrone treatment.

However, not all combinations strategies produced evidence of fully functional CD8⁺ T-cell responses. Yuan et al. [48] observed increased FOXP3⁺ Treg infiltration alongside elevated CD8⁺ T cell counts in early responders and also identified PD-1⁺CD38^hi^ dysfunctional CD8⁺ T cells within the TME. In addition, Zareian et al., [49] measured exhaustion markers PD-1 and TIM-3, degranulation marker CD107a, apoptosis marker CD95, and cytokines IFN-γ, TNF-α, and IL-2 on expanded CD8⁺ T cells following vaccination using multicolour flow cytometry. Combinations involving radionuclide therapy [50] or intensive multimodal treatment [35, 48, 49], were associated with persistent or even increased Treg activity, suggesting that immunoregulatory mechanisms may remain active despite evidence of immune-cell expansion.

## Discussion

Among the included studies, it was observed that monotherapies (Table S2) had shown limited and heterogeneous impacts on Tregs in prostate cancer. They often failed to effectively reduce Treg abundance or function although they enhanced effector T-cell infiltration in certain instances [32, 34–37]. Even though majority of monotherapies had limited success, Maeda et al. & Fujita et al. suggest that success is possible when targeting specific recruitment pathways that interfere with the trafficking or viability of Tregs [28, 31].

In comparison, a stronger ability to overcome Treg-mediated immunosuppression was demonstrated by combination therapy, particularly the ones combined with ADT, chemotherapy, immune-priming agents, or targeted pathway inhibition more often displayed improved CD8⁺ T cell to Treg ratios and enhanced antitumor immune activity [41, 42, 44, 46, 51]. Importantly, the results of these studies indicate that Treg depletion is not enough to trigger long-lasting antitumor immunity. Instead, it seems that therapeutic efficacy will demand a remodelling of the immunosuppressive TME, in which Treg modulation occurs alongside antigen release, inflammatory signaling, and effector T-cell recruitment.

This review’s findings on the limited success of monotherapy aligns with previous systematic studies on immune checkpoints, which have shown inconsistent clinical benefits and low disease control rates in prostate cancer [52, 53]. However, this review builds up on the available findings by directly bringing together the information on Treg regulation and defining immune suppression as a key mechanism limiting the efficacy of monotherapy.

The enhanced immune modulation of combination strategies is consistent with new clinical and translational findings which is in favor of multimodal regimens. For instance, Rosenfeld et al. showed that combinations such as sipuleucel-T plus Ra-223 or Lu-PSMA-617 plus pembrolizumab were associated with stronger clinical and immune responses [54]. Chen et al. also stated that to overcome immunosuppressive factors in the TME, such as Tregs and TGF-β, requires a combination approach that targets many immunosuppressive pathways to boost antitumor immunity which suggest that this effect could be explained by mechanistic synergy [55].

### Why monotherapies underperform in overcoming Treg-mediated immunosuppression

This underperformance can be explained by the multiple overlapping immunosuppressive pathways within the prostate TME, which maintain Treg-mediated suppression despite single-agent intervention. Although one pathway is blocked, other mechanisms such as TGF-β signaling, PD-L1 stabilization, STAT3 activation, and CCR4-mediated Treg trafficking maintains Treg suppressive function [28, 32, 44, 56]. As a result, a decrease in Treg numbers does not always lead to recovery of immune response and in some cases feedback loops actually stimulate Treg expansion.

Although the pathways mentioned above usually often render immunotherapies ineffective, specific targeting of these escape mechanisms has shown promise as a monotherapy. For instance, while Maeda et al. identified CCR4-mediated trafficking as a primary resistance mechanism, their study further demonstrated that direct anti-CCR4 monotherapy was capable of significantly depleting circulating Tregs and improving the CD8/Treg ratio in a canine model [28]. Similarly, the use of HVJ-E demonstrated that viral particles can cause depletion of the Tregs population and accompanied by an increase in the NK cells activity of human patients [31]. These exceptions indicate that monotherapy does not fail in all cases; instead, the success of single agent treatment is determined by the selection of a target that either directly depletes the cells (CCR4) or broadly immunogenically primes (HVJ-E).

### Biological mechanisms underlying Treg modulation in prostate cancer

The studies included explain the biological mechanisms through which combination therapies modulate Tregs and enhance anti-tumor immunity. ADT appears to affect Treg dynamics indirectly by stimulating thymic output, promoting antigen presentation, and effector T-cell infiltration. Preclinical models showed that combining ADT with intratumoral Treg depletion resulted in significant tumor regression and better CD8⁺/Treg ratios than either intervention alone, supporting the role of hormonal manipulation as an immune-priming strategy [41]. Clinical studies also show that while ADT increases overall T-cell infiltration, leftover Tregs may adopt resistance mechanisms to adapt leading to immunosuppression unless additional immune modulation is applied [57].

Immunogenic cell death (ICD) induced by chemotherapy is another method that supports Treg regulation by helping the immune system recognize and attack cancer not just shrinking tumors itself. Oxaliplatin, mitoxantrone, and gemcitabine were demonstrated to stimulate antigen degradation and inflammatory stimulation and make tumours susceptible to immune checkpoint blockade and vaccine-based therapies. Across multiple preclinical studies this immune activation was also linked to reduced Tregs infiltration or dominance within the TME [36, 42, 58].

There were also several preclinical studies that uncovered specific pathways that help maintain Treg immune suppression role, including STAT3 signaling [44], PD-L1 stabilization via USP2 [56] and FLII [30], integrin-mediated activation of TGF-β [32], and Chemokine-dependent Treg trafficking(CCR4 or CCL2) [28, 59]. Interventions targeting these pathways often improved effector T-cell activity without fully depleting Tregs, emphasizing that reprogramming Treg function can be just as important as reducing their numbers.

Moving beyond standard checkpoint blockade, there is need to focus on unconventional pathways to disrupt the suppression of effector cells by Tregs. These pathways identified in preclinical studies, include PRC1-driven CCL2 signaling [59] or FLII-mediated pathways [30]. Also, Shi et al. found that loss of PTEN and TP53 drives Sp1-mediated upregulation of the checkpoint molecule B7-H3 and is associated with Treg enrichment in the prostate TME; combining B7-H3 inhibition with PD-L1 or CTLA-4 blockade overcame this Treg-mediated resistance to achieve durable antitumor responses [37]. This is complemented by the work of Ager et al., who found that STING (Stimulator of Interferon Genes) is an effective means of re-priming the cold prostate TME and favouring the recruitment and activation of T cells [43].

The latest development of delivery systems in preclinical studies has opened new approaches for Treg modulation. Fan et al. used extracellular vesicles derived from mature dendritic cells to deliver cDC1-specific chemokines (XCL1), which enhanced the recruitment of effector cells and overcome the suppressive barriers typically maintained by Tregs [60]. Similarly, Yan et al. used a method of gene-delivery to force the expression of the LIGHT molecule specifically within tumor cells causing those cells to produce anti-cancer signal [45].

**Fig. 4:**
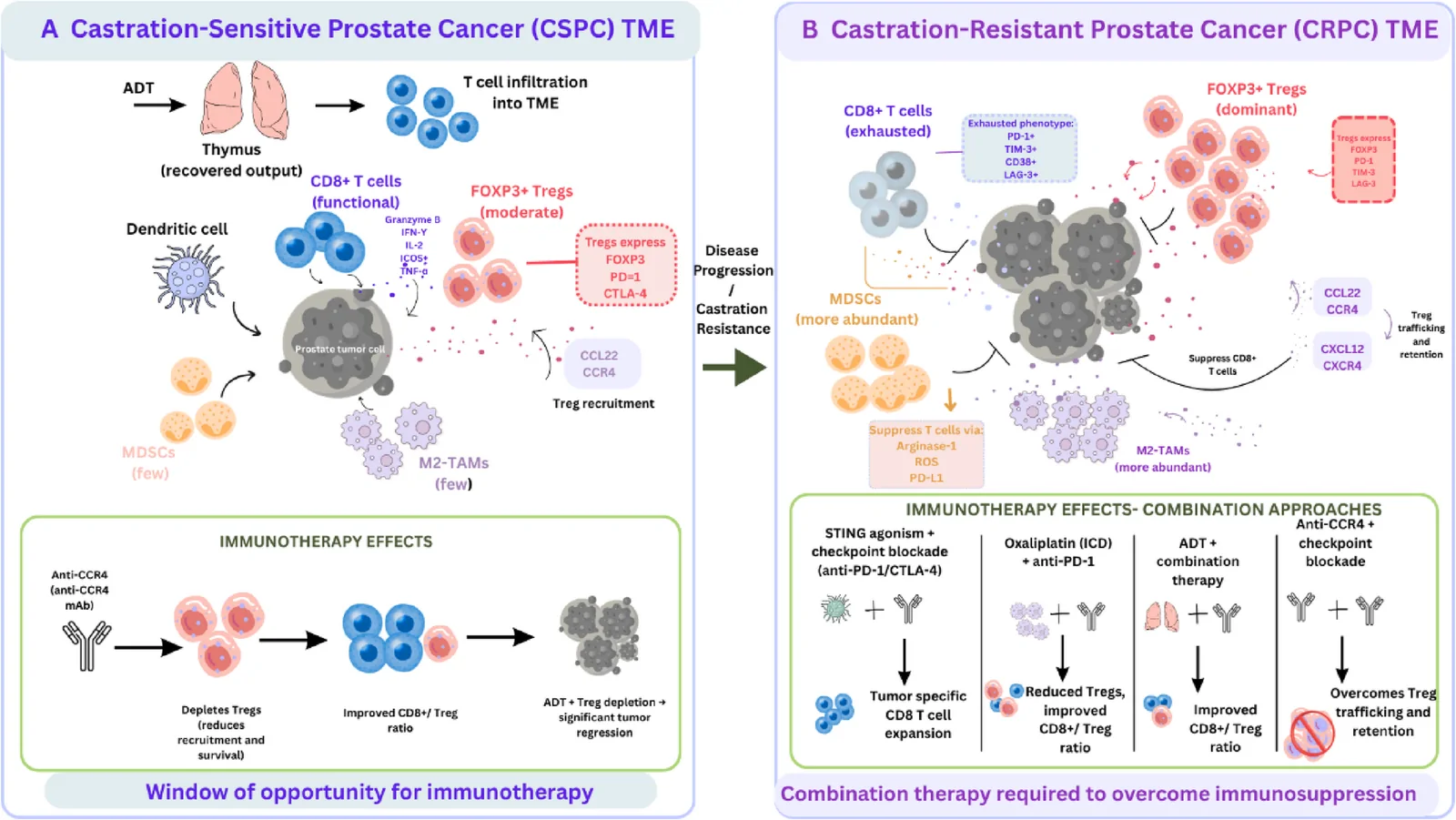
Schematic overview of regulatory T-cell (Treg) modulation during progression from castration-sensitive to castration-resistant prostate cancer. **A** In castration-sensitive prostate cancer (CSPC), androgen deprivation therapy (ADT) promotes thymic recovery and infiltration of functional CD8⁺ T cells while Tregs remain moderately expanded, creating a therapeutic window during which Treg-targeting strategies can enhance antitumor immunity. **B** Progression to castration-resistant prostate cancer (CRPC) is associated with Treg dominance, CD8⁺ T-cell exhaustion, increase in other immunosuppressive populations, and sustained Treg recruitment through the CCL22/CCR4 and CXCL12/CXCR4 pathways, resulting in a highly immunosuppressive tumor microenvironment. Consequently, the figure summarizes the selected immunotherapeutic strategies identified across the included studies that were associated with modulation of Treg-mediated immunosuppression within the prostate TME

Overall, the evidence reviewed highlights that Treg modulation in prostate cancer involves multiple biological mechanisms that collectively shape the tumor microenvironment. Figure 4 summarizes Treg modulation within the evolving tumor immune microenvironment during progression from castration-sensitive to castration-resistant prostate cancer and highlights selected immunotherapeutic strategies identified across the included studies.

### Biomarker implications

While FOXP3^+^ remains the most commonly used Treg marker, its predictive and prognostic function in prostate cancer is ambiguous. FOXP3⁺ Tregs in prostate cancer may sometimes imply increased overall immune activity in individuals who respond to therapy. Clinical findings of Yuan et al. & van Dodewaard et al. show that Tregs increased along with high populations of CD8 + T cells, resulting in an optimal CD8 ^+^-to-Treg ratio and general immune activation. In these instances, the increase does not imply treatment failure but immune activation or a warmed TME [47, 48].

This underscores the need to switch to integrated immune profiling that records functional shifts in the TME. For instance, preclinical findings by Potluri et al. showed that targeted radionuclide therapy when combined with anti-PD-1 can inadvertently trigger Treg proliferation, creating an immunosuppressive barrier that limits therapeutic efficacy [50]. Without detailed profiling, this proliferation might be misinterpreted as general activation. In contrast, clinical findings from Zareian et al., demonstrated the value of integrated profiling by using TCR sequencing, a molecular fingerprinting method to track the expansion of hTERT-specific T- cells clones [49]. Their findings revealed that even when Treg-modulating agent cyclophosphamide failed to significantly reduce Treg frequencies, the vaccine still induced durable clinical benefit. Preclinical research also highlights genetic biomarkers such as PTEN status as indicators of the suppressive nature of the TME. Shi et al. showed that PTEN/TP53 inactivation serves as a molecular driver for Treg enrichment [37], whereas Lin et al. showed that restoring PTEN expression can effectively dismantle this immunosuppression [26].

### Clinical implications

The summary of this data (Table S1) shows that Treg-targeted treatments are unlikely to be successful as stand-alone therapies for prostate cancer. Instead, well- designed combination therapies that include androgen deprivation, chemotherapy, radiotherapy, or immune-priming agents may better reprogram the prostate TME from immunologically “cold” to “hot”.

It is important to note that Treg counts alone should not be interpreted as uniformly negative because Treg numbers may increase in responders while the CD8⁺/Treg ratio improves, and evidence of immune activation is observed, suggesting that ratios and functional status are more informative than total abundance. Therefore, metrics such as effector-to-suppressor ratios and pathway-specific markers may be a better biomarker for treatment selection and monitoring compared with FOXP3⁺ Treg counts alone [47, 48].

The findings here also suggest significant shifts in the treatment of prostate cancer. Clinical evidence shows that monotherapies given without first preparing the TME routinely fail to overcome Treg-mediated suppression; personalized vaccines showed minimal impact on the TME when used alone [34, 35]. Preclinical evidence, however, points to several molecular strategies for priming the TME that may sensitize tumors to immunotherapy, pending clinical validation. These include combining checkpoint inhibitors with other agents rather than using them alone [36], priming with oxaliplatin to induce immunogenic cell death [42] or ADT to boost effector T-cell infiltration [41], and directly targeting mechanisms of immune evasion such as PRC1 inhibition combined with dual checkpoint blockade [59] and USP2 depletion + PD-1 inhibition [56] both resulted in significant Treg reduction and restored T-cell cytotoxicity. These strategies represent promising candidates for future clinical investigation.

Beyond changes in Treg frequency, an important consideration across the combination therapy studies included in this review is the functional state of CD8⁺ T cells. While several studies reported increased CD8⁺ T-cell infiltration or improved CD8⁺/Treg ratios following treatment, these findings did not always correspond to the same level of functional activity. The strongest mechanistic evidence comes from preclinical studies. For example, Shen et al. [41] demonstrated active cytotoxic CD8⁺ T-cell responses during the peri-castration period, with increased production of granzyme B, IFN-γ, TNF-α, and IL-2. Interestingly, this was accompanied by elevated expression of the exhaustion markers PD-1, CTLA-4, LAG-3, and TIM-3, which later declined as castration resistance developed. Similarly, Ager et al. [43] showed that STING agonism combined with checkpoint blockade expanded tumor-reactive CD8⁺ T cells, as identified by SPAS-1 tetramer staining, and was associated with increased TCR repertoire diversity.

Clinical studies, however, present a more cautionary picture. Zareian et al., [49] reported that CD8⁺ T cells expanded following hTERT vaccination co-expressed activation markers (CD107a and HLA-DR) alongside the exhaustion markers PD-1 and TIM-3 and the apoptosis marker CD95. Likewise, Yuan et al. [48] identified dysfunctional PD-1⁺CD38^hi^ CD8⁺ T cells within the tumor microenvironment, suggesting that increased immune-cell infiltration does not necessarily translate into fully restored cytotoxic function.

Taken together, these findings indicate that increases in CD8⁺ T-cell infiltration or improvements in CD8⁺/Treg ratios should be interpreted with caution, as they may not always reflect effective antitumor immunity. Notably, the clearest evidence regarding the timing and reversibility of CD8⁺ T-cell exhaustion comes from preclinical models, whereas clinical studies currently provide more limited, but direct, evidence of exhausted T-cell phenotypes in patients.

### Limitations

When evaluating these findings, a number of limitations must be considered. First, many included studies were preclinical, with murine or other animal models that may not completely recapitulate the complex human prostate cancer immunobiology. The clinical studies were frequently limited by small sample sizes, early-phase designs, and heterogeneous patient populations, all of which reduced the precision and generalizability of the findings. Another important limitation was the high risk of confounding within the non-randomized studies. All eight non-randomized clinical studies showed serious risk of bias in the confounding domain, and none used modern confounding-control approaches such as propensity score matching (PSM) or inverse probability of treatment weighting (IPTW) or multivariable Cox regression to account for baseline differences between study groups. Podrazil et al. represented a partial exception, as they employed univariate and multivariate Cox proportional hazards analyses to adjust for relevant clinical covariates. Consequently, some of the reported differences in Treg frequency or function may have been influenced by underlying patient characteristics, including metastatic burden, disease stage, or prior exposure to androgen deprivation therapy (ADT), rather than reflecting treatment-induced immunological effects alone.

Second, there was significant variation in how Tregs were defined and measured. The use of markers like FOXP3^+^, CD4^+^CD25^+^ or functional assays was not used consistently, and not many studies identified Treg subsets or made an evaluation of suppressive function as well as frequency. Third, variability in treatment regimens, dosing schedules, and timing of immune sampling limited direct comparison across studies. Additionally, none of the included studies evaluated TOX or TCF7 expression, transcription factors commonly associated with terminal CD8⁺ T-cell exhaustion, representing a gap in the current evidence base for fully characterizing CD8⁺ T-cell functional states across both preclinical and clinical settings. Lastly, the review was limited to English-language papers from the last 10 years as well as database searching, which may have excluded potential relevant unpublished or non-indexed studies.

## Conclusion

Future research should focus on the development of well-designed clinical trials which include comprehensive immune profiling prior to, during, and following therapy. To better explain their predictive and prognostic significance, Treg phenotyping needs to be standardized, including distinguishing between effector, resting, and memory-like Treg subsets and evaluating suppressive activity. Studies must not limit their analysis to counting of Tregs to assessing functional reprogramming, resistance mechanisms, and interactions with other immune and stromal populations.

Combination treatment, especially those that combine immune checkpoint inhibition with agents that enhance antigen release, promote T-cell priming, or disrupt Treg recruitment and survival should be further looked into. Concurrently, the creation of composite biomarkers that include pathway-specific markers, cytokine signatures, and CD8/Treg ratios may facilitate individualized treatment choices. The longitudinal sampling and association with clinical outcomes including PFS and OS will be important in establishing whether Treg modulation is a cause of benefit or a simple by-product of global immune activation.

## Supplementary Information

Supplementary Material 1.

## Data Availability

All data generated and analysed during this study are included in this published article.

## Abbreviations

ADT: Androgen Deprivation Therapy
APC: Antigen-Presenting Cell
CD: Cluster of Differentiation
CTLA-4: Cytotoxic T-Lymphocyte-Associated Protein 4
FOXP3^+^: Forkhead Box P3
ICI: Immune Checkpoint Inhibitor
PD-1: Programmed Cell Death Protein 1
PD-L1: Programmed Death-Ligand 1
PSA: Prostate-Specific Antigen
TME: Tumor Microenvironment
Treg: Regulatory T cell
