# Supplementary material for: Impact of immunotherapy as a monotherapy or in combination with conventional therapies on regulatory T-cell (Treg) modulation in prostate cancer: a systematic review
Source: BMC Cancer. 2026 Jul 17;26:1233. doi: 10.1186/s12885-026-16550-w (PMC13670563; doi:10.1186/s12885-026-16550-w)
Supplement: Supplementary file 1 [file 12885_2026_16550_MOESM1_ESM.docx]

**SUPPLEMENTARY MATERIALS**

|  | Table S1: Types of the included studies and their results. This table outlines the authors, publication year, study types, and study outcomes. | | | |
| --- | --- | --- | --- | --- |
| **Authors & Year** | | **Type of Study** | **Result of the Study** |  |
| Shen et al., 2018 [41] | | Preclinical study | Anti-CD25 (Treg depletion) combined with ADT enhanced CD8: Treg ratio and delayed castration resistance. |  |
| Zhou et al., 2017 [42] | | Preclinical study | Oxaliplatin increased tumor immunogenicity and improved responsiveness to immune checkpoint blockade. |  |
| Witt et al., 2021[44] | | Preclinical study | Blocking STAT3 amplified anti-CTLA-4 therapeutic efficacy and strengthened T-cell–mediated antitumor responses. |  |
| Li et al., 2025 [58] | | Preclinical study | ZIF-8 nanocarrier delivering Mitoxantrone immunogenic cell death and improved chemo-immunotherapy outcomes. |  |
| Lin et al., 2021 [26] | | Preclinical study | PTEN restoration via mRNA nanoparticles increased antitumor immunity and suppressed tumor progression. |  |
| Potluri et al., 2022 [49] | | Preclinical study | Therapy was effective as TDT but limited by expansion of regulatory T cells occurring as a result of Anti- PD-1. |  |
| Anker et al., 2018[27] | | Preclinical study | A clinical bacterial isolate modulated immunity and enhanced antitumor immunotherapy efficacy. |  |
| Obradovic et al., 2020 [57] | | Clinical trial | ADT increased T-cell infiltration; vaccination induced adaptive Treg resistance mechanisms. |  |
| Maeda et al., 2022 [28] | | Preclinical study | The Anti-CCR4 therapy effectively depleted Tregs and demonstrated increasing survival benefits in canine model. |  |
| Guo et al., 2021 [29] | | Preclinical study | The fusion vaccine induced strong and specific immune responses that restricted prostate tumor growth. |  |
| Wei et al., 2016 [38] | | Clinical translational study | Admission of GM-CSF resulted in recruitment of effector T cells into the tumor microenvironment, increasing antitumor immunity. |  |
| Dodagatta-Marri et al., 2021[32] | | Preclinical study | Blocking αvβ8 on Tregs prevent the activation of TGF-β, which reactivates CD8+ T cells increasing Granzyme B/IFN-γ production |  |
| Shi et al., 2023 [37] | | Preclinical study | B7-H3 inhibitor alone in PTEN/TP53-deficient models was hindered by enriched intratumoral Tregs, but combinations with PD-L1/CTLA-4 blockade was required to overcome immunosuppression. |  |
| Galsky et al., 2025 [51] | | Phase I Clinical Trial | The objective of the trial confirmed that clinical benefit was associated with favorable immune modulation, including reduction of Tregs in the tumor. |  |
| Su et al., 2019 [59] | | Preclinical study | PRC1 drove metastasis and promoted immune suppression in DNPC by inducing CCL2-mediated recruitment of Tregs, inhibiting PRC1 reversed this immune evasion and sensitized tumors to dual checkpoint blockade. |  |
| Podrazil et al., 2015 [46] | | Phase I/II Clinical Trial | Combination of dendritic-cell vaccine (DCVAC) with chemotherapy was safe and showed enhanced immune activation with clinical benefit trends in mCRPC. |  |
| Farsaci et al., 2016 [39] | | Translational clinical study | Vaccine therapy decreased circulating Tregs. Higher baseline peripheral immunoscore correlated with improved immune response and clinical outcomes. |  |
| Kuang et al., 2023 [56] | | Preclinical study | USP2 promoted tumor immune evasion by deubiquitinating and stabilizing PD-L1, enhancing Treg-mediated suppression. |  |
| Yan et al., 2015 [45] | | Preclinical study | Forced LIGHT expression reversed Treg-mediated suppression and synergized with therapeutic vaccine by boosting effector T-cell infiltration. |  |
| Johnson et al., 2017 [40] | | Translational clinical study | High baseline levels of antigen-specific Tregs predicted a poorer immune response |  |
| Ruan et al., 2021 [30] | | Preclinical study | Flightless I reversed enzalutamide resistance by inhibiting YBX1/PD-L1 pathway suppressed Tregs and restored antitumor immunity. |  |
| Fujita et al., 2017 [31] | | Phase I/II Clinical Trial | Intratumoral and subcutaneous HVJ-E therapy induced immune stimulation with antitumor activity. |  |
| Ager et al., 2017 [43] | | Preclinical study | Intratumoral STING agonism combined with checkpoint inhibitors generated strong systemic antitumor immunity. |  |
| Noguchi et al., 2016 [34] | | Randomized Phase II Clinical Trial | Combination reduced circulating Tregs, but failed to improve OS or PFS. |  |
| Fan et al., 2025 [60] | | Preclinical study | XCL1-loaded DC–derived EVs enhanced chemotherapy-induced ICD and boosted CD 8+ T cell infiltration. |  |
| Gasparri et al., 2025 [33] | | Preclinical study | Blocking αvβ6/αvβ8 inhibited TGFβ activation, reduced immune suppression by inhibiting Treg function and activity |  |
| Koga et al., 2017 [35] | | Translational clinical study | The herbal formulation combined with PPV did not alter Treg frequency as observed with vaccine therapy alone. |  |
| Ariyan et al., 2018 [36] | | Preclinical study | Chemotherapy when combined with CTLA-4 blockade, induced immunogenicity and robust antitumor immune responses and significantly reducing Treg, whereas anti-CTLA-4 alone was insufficient. |  |
| Yuan et al., 2021 [48] | | Phase I clinical trial | Combination therapy was feasible and safe; exploratory analyses showed immune activation and early signals of therapeutic benefit even with the increase in Tregs. |  |
| van Dodewaard‐de Jong et al., 2016 [47] | | Clinical translational study | GVAX + ipilimumab improved mitoxantrone efficacy, enhanced immune activation, a high effector-to-Treg ratio improved progression-free survival (PFS). |  |
| Zareian et al., 2024 [50] | | Phase I clinical trial | hTERT vaccination combined with immune-modulators enhanced enhanced antigen-specific T cell responses and clinical stability, although limited by T-cell exhaustion and lack of significant Treg depletion. |  |

Table S2: Effects of Immunotherapy Monotherapies on Treg Cells in Prostate Cancer. Note: ↑ indicates increase; ↓ indicates decrease; ↔ indicates stability of Treg cells.

| Study | Intervention | Population | Treg Outcome | Direction | Notes |
| --- | --- | --- | --- | --- | --- |
| Lin et al., 2021 [26] | PTEN mRNA Nanoparticles | Preclinical models (Pten-null prostate cancer cells) | Reduced Treg cells and MDSCs in the TME. | **↓** | PTEN restoration reversed the immunosuppressive TME, promoted CD8^+^ T cell infiltration, and enhanced proinflammatory cytokines. |
| Anker et al., 2018 (Monotherapy arm) [27] | Clinical Bacterial Isolate (CP1) administered intra-urethrally | Orthotopic preclinical models | Decreases intra-tumoral regulatory T cells and VEGF. | **↓** | CP1 bacteria isolate primes the microenvironment and causes, depletion of Tregs |
| Maeda et al., 2022 [28] | Anti-CCR4 monoclonal antibody (Mogamulizumab) | Dogs with naturally occurring advanced prostate cancer | Decreased circulating Tregs and improved the CD8+ Treg ratio. | **↓** | CCL17-CCR4 is highly expressed on Tregs, and identified as primary driver of Treg infiltration. Anti-CCR4 shows capacity of depleting circulating Tregs while increasing CD8+ cells |
| Guo et al., 2021 [29] | Fusion Protein Vaccine (Ag85B-STEAP1) | Preclinical mouse model (Prostate cancer cell line RM-1) | Decreased percentages of Tregs in the spleens and tumor-draining lymph nodes | **↓** | The Ag85B-STEAP1 fusion vaccine induced robust antigen-specific CD8+ T-cell and antibody responses. |
| Wei et al., 2016 [38] | Systemic GM-CSF (Granulocyte-Macrophage Colony-Stimulating Factor) | Phase 1 human study: Localized prostate cancer patients receiving neoadjuvant treatment. | Increased circulating FoxP3^+^ CD4^+^ regulatory T cells, but to a lesser magnitude than effector T cells. | **↑** (Minor) | CD8 ^+^T cell was recruited into the tumor. The increase in Tregs observed was less pronounced than the increase in effector T cells and dendritic cells. |
| Dodagatta-Marri et al., 2021 [32] | Anti-αvβ8 monoclonal antibody (ADWA-11) | Syngeneic mouse model (TRAMPC2 prostate cancer) | Treg suppressive function blocked (due to TGF-β inhibition). | **↔** | The monoclonal antibody acts by inhibiting the αvβ8 integrin on CD4^+^ CD25^+^ T cells (which includes Tregs), thereby preventing the activation of immunosuppressive TGF- β. Cell quantity was not reported to be depleted. |
| Shi et al., 2023 [37] | B7-H3 Inhibitor (MJ18) | Preclinical model (Pten/Tp53-deficient CRPC GEMM) | Accumulation of regulatory T cells (Tregs) in tumors. | **↑** | The accumulation of Tregs and elevated PD-L1 on myeloid cells hindered the efficacy of the B7-H3 inhibitor monotherapy, supporting the need for combination therapy. |
| Farsaci et al., 2016 [39] | Vaccine Therapy | Human study: mCRPC patients enrolled in a Phase 2 trial. | Decreased circulating Tregs (CD4^+^ CD25 ^high^ FoxP3^+^) following vaccination in patients who had a positive overall immune response. | **↓** | Study found that a low pre-therapy circulating immune score, driven by high CD4^+^ Tregs, predicted poor response to the vaccine. Successful vaccination was associated with Treg decrease. |
| Johnson et al., 2017 [40] | DNA Vaccine (Targeting PAP) | Human study: Non-metastatic and CRPC patients in Phase 1 trials. | Elevated antigen-specific Tregs pre-treatment correlated with poorer subsequent immune response to vaccination. | **↑** | The presence of regulatory cells (Tregs) specific to the vaccine target before treatment inhibits the development of a therapeutic CD8 ^+^ T cell response. |
| Ruan et al., 2021 [30] | FLII Restoration (Genetic or pharmacological restoration of Flightless I Homolog) | Preclinical model (Enzalutamide-resistant PCa) | Inhibition of Treg infiltration in the tumor. | **↓** | Restoration of FLII reversed resistance by inhibiting the YBX1/PD-L1 pathway, thereby activating anti-tumor T-cell responses and inhibiting Treg recruitment. |
| Fujita et al., 2017 [31] | HVJ-E (Inactivated Sendai Virus Particles) | Phase I/II human study: CRPC patients (Docetaxel-resistant or ineligible). | Suppression of regulatory T cells population in vivo | **↓** | Clinical trial showed safety and elevated NK cell activity. The anti-tumor mechanism of HVJ-E includes Treg suppression. |
| Gasparri et al., 2025 [33] | Chromogranin A-derived peptide (targeting integrins αvβ6/αvβ8) | Preclinical models (PC-3 xenograft and MMTV-PyMT mouse models) | Treg suppressive function inhibited via TGF-β pathway blockade. | **↔** | The peptide acts by blocking TGF-β activation, which is necessary for Treg-mediated immune suppression and tumor growth. The cell quantity itself was not the primary outcome. |
| Noguchi et al., 2016 (Monotherapy Arm) [34] | Personalized Peptide Vaccination (PPV) alone | 34 patients with metastatic castration-resistant prostate cancer (mCRPC | Mean frequencies of circulating Tregs showed no significant change, moving from 0.94 ± 1.0 at pre-treatment to 0.8 ±0.77% at 7 weeks (p = 0.84) | **↔** | PPV as a monotherapy failed to significantly alter the circulating Treg population in mCRPC patients. |
| Koga et al., 2017 (Monotherapy Arm) [35] | Personalized Peptide Vaccination (PPV) alone | 35 patients assigned; 31 analyzed for immune response | There was no significant change in the frequency of circulating regulatory T cells (CD4^+^CD25^+^FoxP3^+^)3 | **↔** | In this arm, while Tregs remained stable, the study found a significant increase in both monocytic myeloid-derived suppressor cells (Mo-MDSCs) (p=0.012) and IL-6 levels (p=0.04) during treatment. |
| Ariyan et al., 2018 (Monotherapy Arm) [36] | Anti-CTLA-4 alone | TRAMPC2 Murine Model (Prostate Cancer) | Monotherapy treatment had only a modest effect on tumor growth and failed to robustly alter the suppressive environment | **↔** | In this prostate cancer model, Anti-CTLA-4 was insufficient to trigger the necessary inflammatory shift to overcome immune suppression. |

TABLE S3: Effects of Combination therapies on Treg Cells in Prostate Cancer. Note: ↑ indicates increase; ↓ indicates decrease; ↔ indicates stability.

| Study | Intervention | Population | Treg Outcome | Direction | Notes |
| --- | --- | --- | --- | --- | --- |
| Shen et al., 2018 [41] | Intratumoral Treg Depletion (Anti-CD25 antibody) + ADT (LHRH agonist) | Preclinical model (Myc-CaP PCa) | Intratumoral Treg depletion was augmented by ADT. | **↓** | ADT alone caused a transient increase in CD8 ^+^ T cells and enhanced the effect of the depletion agent. The combination resulted in significant tumor regression and improved CD8^+^/ Treg ratio. |
| Zhou et al., 2017 [42] | Oxaliplatin (Chemotherapy) + Immune Checkpoint Blockade (Anti-PD-1/Anti-CTLA-4) | Preclinical model (TRAMP-C2 PCa) | Combination with Oxaliplatin reduced Tregs in the TME. | **↓** | Oxaliplatin induced immunogenic cell death (ICD) switching the cold TME to a hot one, which sensitized the tumor to the checkpoint blockade. |
| Witt et al., 2021 [44] | STAT3 Inhibitor + Anti-CTLA-4 Checkpoint Blockade | Preclinical model (Prostate cancer cell lines) | The combination strategy significantly reduced the number of Tregs and MDSCs in the tumor. | **↓** | STAT3 inhibition reversed the immunosuppressive TME by preventing the rise of IL-10, resulting in increased CD8^+^ T-cell infiltration and extended survival benefit with CTLA-4 blockade. |
| Li et al., 2025 [58] | Mitoxantrone (Chemotherapy in ZIF-8 Nanocarrier) + Anti-CTLA-4 | Preclinical model (Prostate cancer cell lines RM-1and 4T1) | The treatment significantly reduced the percentage of Tregs in the tumors. | **↓** | The nanoplatform delivered Mitoxantrone to induced pyroptosis and depleted Tregs as a monotherapy. When combined with anti-CTLA-4 led to increased anti tumor responses |
| Potluri et al., 2022 [49] | Combined Therapy: Targeted Radionuclide Therapy ^90^ Y-NM600 + PD-1 blockade | Murine prostate tumors (preclinical model) | Tregs proliferate and maintain an immunosuppressive environment that limits the anti-tumor efficacy of the combined therapy. | **↑** | The Targeted Radionuclide Therapy primed the TME, causing infiltration and increase of CD8+ T cells and increased PD-L1 expression. Adding the anti-PD-1 drug as combination activated Tregs |
| Obradovic et al., 2020 [57] | Combined Therapy (ADT + Prostate Cancer Vaccination) | Localized Prostate Cancer (PCa) patients | ADT increases T-cell infiltration, but remaining Tregs exhibit "adaptive resistance" (increased function), maintaining immunosuppression | **↑** | This adaptive resistance creates a loop where the treatment's effectiveness gets suppressed and limits the clinical efficacy of the vaccine. |
| Galsky et al., 2025 [51] | Multiple combinations (Checkpoint Inhibitors + Costimulatory Agonists/Targeted Agents) | Phase I human trial: mCRPC patients | The combination therapies successfully modulated the TME, leading to decreased Treg suppression in responders. | **↓** | Nivolumab-based combinations (particularly with radiotherapy and CDX-301) showed clinical activity |
| Podrazil et al., 2015 [46] | Combined Therapy: Dendritic-Cell based Immunotherapy (DCVAC/PCa) + Chemotherapy (Docetaxel) | Phase I/II human trial: mCRPC Pca patients | The combination therapy resulted in decreased numbers of circulating Tregs in the peripheral blood of treated patients. | **↓** | This clinical trial demonstrated that combining an active immune-stimulatory agent (DCVAC/PCa) with chemotherapy can successfully reduce the systemic immunosuppressive burden (↓ Tregs), suggesting a favorable shift in immune balance. |
| Yan et al., 2015 [45] | Gene Therapy (Forced LIGHT Expression) + Therapeutic Prostate Tumor Vaccine | Preclinical model (Murine prostate cancer) | Forced expression of the LIGHT molecule in tumor cells was shown to overcome Treg-mediated immunosuppression and synergize with the tumor vaccine. | **↓** | The direction indicates Treg-mediated suppression (via LIGHT molecule signaling), which recruits effector T lymphocytes and enhances the efficacy of the vaccine. |
| Ager et al., 2017 [43] | Intratumoral STING Activation + T-cell Checkpoint Modulation (Anti-PD-1) | Preclinical model (Murine prostate tumors) | The combination resulted in a robust anti-tumor response associated with decreased infiltration of Tregs and Myeloid-Derived Suppressor Cells (MDSCs) in the tumor. | **↓** | STING activation induced immunogenic cell death and Type I IFN release, reversing the immunosuppressive tumor microenvironment and generating systemic (abscopal) immunity. |
| Noguchi et al., 2016 (Combination arm) [34] | Personalized Peptide Vaccination (PPV) + Metronomic low-dose Cyclophosphamide (CPA, 50 mg/day) | 70 patients with metastatic castration-resistant prostate cancer (mCRPC) | The combination led to a significantly greater percentage (%) decrease in circulating Tregs compared to the monotherapy group (-15.3% vs. +87.6%, p = 0.036) | **↓** | While CPA successfully reduced Tregs, it also caused a simultaneous increase in MDSCs, which suggest may a neutralized antitumor effect, as no significant difference in Overall Survival (OS) or Progression-Free Survival (PFS) was observed between the two arms. |
| Fan et al., 2025 [60] | XCL1-loaded DC-derived Extracellular Vesicles (DEX _XCL1_) + Cisplatin-induced Immunogenic Cell Death (ICD) | Preclinical mouse models of CRPC (RM-1 and OVA-expressing RM-1 cells) | The combination therapy significantly reduced the proportion of regulatory T cells (Tregs) within the tumor tissue. | **↓** | This therapy successfully reshaped the immunosuppressive TME. DEX _XCL1_ enhanced the recruitment and activation of cDC1 cells, while cisplatin-induced ICD released tumor-associated antigens (STEAP1), resulting in increased CD8 ^+^ T-cell infiltration and tumor suppression. |
| Koga et al., 2017 (Combination Arm) [35] | Herbal medicines (Hochu-ekki-to and Keishi-bukuryo-gan) + Personalized Peptide Vaccination (PPV) | 31 patients treated; 26 analyzed for immune response | Like the monotherapy arm, there was no significant change in the frequency of circulating Tregs following the combination treatment (p = 0.84). | **↔** | Although Tregs were not impacted, the herbal medicines successfully stabilized both Mo-MDSCs (p=0.96) and IL-6 levels (p=0.63), preventing the immunosuppressive increase seen in the monotherapy group. |
| Ariyan et al., 2018 (Combination Arm) [36] | Gemcitabine + CTLA-4 blockade (anti-CTLA-4) | TRAMPC2 Murine Model (Prostate Cancer) | The combination resulted in fewer Foxp3^+^ Treg cells and a significantly increased CD8/Treg ratio within the TME | **↓** | The synergy between chemotherapy (which induces local inflammation) and checkpoint blockade induced a shift in the TME, favoring effector cells over suppressive Tregs. |
| Yuan et al., 2021 [48] | Nivolumab (Anti-PD-1) + HDR Brachytherapy + EBRT + Androgen Deprivation Therapy (ADT) | 12 patients with Grade Group 5 (GG5) high-risk prostate cancer | Increased levels of FOXP3^+^ CD4^+^ T cells were observed in the tumor tissue of early responders | **↑** | The increase in Tregs occurred alongside a significant increase in CD8+ effector cells, suggesting a total immune "warming" of the TME that correlated with antitumor activity. |
| van Dodewaard et al., 2016 [47] | GVAX (GM-CSF vaccine) + Ipilimumab (anti-CTLA-4) followed by Mitoxantrone (Chemotherapy) | 28 patients with mCRPC (Phase I trial) | Patients with lower pre-treatment rates of Tregs and MDSCs, combined with a high CD8+ICOS+ T cell/Treg ratio, had significantly longer PFS. | **↓ (Rel.)** | A high ratio of effector cells to suppressive Tregs (>1) was strongly associated with a better response to subsequent chemotherapy. |
| Zareian et al., 2024 [50] | hTERT peptide vaccine + Low-dose Cyclophosphamide (CPA) + Celecoxib + Adjuvants (Montanide/Imiquimod) | 5 of 29 patients with prostate cancer | The study found no significant change in the frequency or phenotype of Tregs following the combination treatment (p > 0.05). | **↔** | The trial used metronomic CPA specifically to modulate Tregs, a numerical reduction was observed but failed to reach statistical significance. However, the patients achieved stable disease state for over 6 months. |
| Anker et al., 2018 (combination therapy arm) [27] | CP1 + Anti-PD-1 Checkpoint Inhibitor | Orthotopic preclinical models | Further decreased Tregs and significantly enhanced anti-tumor immunity compared to monotherapies. | **↓** | CP1 isolate primes the environment, depleting Tregs and recruiting effector cells, while anti-PD-1 capitalizes on the hot immune environment to achieve significant tumor regression. |
| Su et al., 2019 [59] | PRC1 Inhibitor (GW-516) + Anti-PD-1 + Anti-CTLA-4 | Preclinical models (Double-Negative Prostate Cancer) | PRC1-driven gene signature was positively correlated with immunosuppressive markers like FoxP3^+^ (Treg marker) in human tumors. | **↓** | Study identifies PRC1 as a driver of immune suppression (by recruiting Tregs) and metastasis in aggressive prostate cancer, linking it to Treg presence. PRC1 inhibition primes the TME, allowing checkpoint blockade to suppress metastasis. |
| Kuang et al., 2023 [56] | USP2 Depletion + Anti-PD-1 | Preclinical models and human samples (PCa) | USP2 depletion reduced Treg survival and suppressive function by destabilizing PD-L1. | **↓** | USP2 stabilizes PD-L1, its removal causes ER-associated degradation of PD-L1, sensitizing cells to T-cell killing and reducing Treg-mediated evasion. |

Table S4. Comprehensive baseline data of included studies

| **Study ID** | **Study Design** | **Intervention Regimen** | **Treg Markers** | **Tumour Model/Stage** | **Sample size** | **Follow-up / Duration** |
| --- | --- | --- | --- | --- | --- | --- |
| Shen et al., 2018 | Murine (FVB/NJ) | ADR (Degarelix) + Anti-CTLA-4 (IgG2a) | CD4^+^FoxP3^+^ | Myc-CaP Prostate Cancer | 5–10 mice per group | 8 weekly injections (Cycle 1) followed by 8 doses at 2-3 week intervals |
| Zhou et al., 2017 | Murine (C57BL/6) | Oxaliplatin + Anti-PD-1 | CD4^+^ CD25^+^ FoxP3^+^ | TRAMP-C2 PCa | 5 mice per group | ~18 days (tumor growth monitoring) |
| Witt et al., 2021 | Murine (C57BL/6) | STAT3 Inhibitor (GPB730)+ Anti-CTLA-4 | CD4^+^ CD25^+^ FoxP3^+^ | RM-1 (Prostate Cancer) | 8–10 mice per group | ~25–30 days (survival tracking) |
| Li et al., 2025 | Murine (C57BL/6) | Mitoxantrone (Chemotherapy in ZIF-8 Nanocarrier) + Anti-CTLA-4 | CD4^+^ FoxP3^+^ | RM-1 (Prostate Cancer) | 5 mice per group | ~20 days (tumor growth) |
| Lin et al., 2021 | Murine (C57BL/6) | PTEN mRNA Nanoparticles | CD4^+^ FoxP3^+^ | Pten-null RM-1 (Prostate Cancer) | 5–8 mice per group | ~20 days (tumor growth monitoring) |
| Potluri et al., 2022 | Murine (C57BL/6) | Radionuclide Therapy ^90^ Y-NM600 + Anti-PD-1 ± Anti-CD25 | CD4^+^ CD25^+^ FoxP3^+^ | RM-1 and TRAMP-C2 | 5–10 mice per group | ~30–60 days (survival) |
| Anker et al., 2018 | Murine (C57BL/6) | 1. Clinical Bacterial Isolate (CP1) 2. CP1 + Anti-PD-1 Checkpoint Inhibitor | CD4^+^ CD25^+^ FoxP3^+^ | Orthotopic MYC/PTEN-mutant PCa | 10 mice per group | ~30 days |
| Obradovic et al., 2020 | Randomized Phase II Clinical Trial | Degarelix (ADT) ± Cyclophosphamide/GVAX vaccine | FoxP3^+^, CD4^+^, CD25^+^, CD127^low^ | Localized Prostate Cancer (PCa) patients | 29 patients | ~4 weeks (pre-prostatectomy) |
| Maeda et al., 2022 | Canine model | Anti-CCR4 (Mogamulizumab) | FoxP3^+^, CD4^+^, CD25^+^ | Spontaneous Advanced Prostate Cancer | 8 Dogs | 4–12 weeks |
| Guo et al., 2021 | Murine (C57BL/6) | Fusion Protein Vaccine (Ag85B-3 × STEAP1) | FoxP3^+^ | RM-1 and TRAMP-C1 | Not specified | ~20 days (tumor growth) |
| Wei et al., 2016 | Phase II Clinical Trial | Systemic GM-CSF | CD4^+^ CD25^+^ CD127^low^ FoxP3^+^ | Localized prostate cancer patients | 22 patients | ~4 weeks (pre-prostatectomy) |
| Dodagatta-Marri et al., 2021 | Murine (C57BL/6) | Anti-αvβ8 (ADWA-11) ± Radiotherapy/Immunomodulators | CD4^+^, CD25^+^ | TRAMPC2 | Not specified | ~30–40 days |
| Shi et al., 2023 | Murine (C57BL/6 and BALB/c) | Anti-B7-H3 ± Anti-PD-1 / Anti-PD-L1 / Anti-CTLA-4 | CD4^+^FoxP3^+^ | PTEN/TP53-deficient Prostate Cancer (mCRPC) | Not specified | ~60 days (Survival tracking) |
| Galsky et al., 2025 | Phase I Clinical Trial (mCRPC, multicohort) | Cohort A: Bempegaldesleukin + Nivolumab;  Cohort B: SBRT + CDX-301 + Poly-ICLC + Nivolumab;  Cohort C: CDX-301 + INO-5151 + Nivolumab | General Phenotype | Phase I human trial: Metastatic CRPC patients | 43 patients | Up to 2 years of treatment |
| Su et al., 2019 | Murine (C57BL/6) | PRC1 Inhibitor (GW-516) ± Anti-PD-1 + Anti-CTLA-4 | CD4^+^CD25^+^FoxP3^+^ | Double-negative Prostate Cancer (DNPC) | Not specified | Varied Metastasis tracking |
| Podrazil et al., 2015 | Open-label, single-arm Phase I/II clinical trial | DCVAC/PCa (dendritic cells pulsed with killed LNCAP) + Metronomic cyclophosphamide (50 mg) + Docetaxel + Prednisone | CD4^+^CD25^+^FoxP3^+^ | Progressive mCRPC | 25 patients | One-year treatment period |
| Farsaci et al., 2016 | Translational clinical / correlational study | Quadramet (^153^ *S*m) + PROSTVAC vaccine vs. Quadramet alone | FoxP3^+^CD4^+^, ICOS^+^ | Human study: mCRPC patients enrolled in a Phase 2 trial. | 44 men with androgen-insensitive (castration-resistant) Pca and metastatic bone lesions. | Quadramet every 12 weeks; PROSTVAC on days 1, 15, 29, then every 4 weeks |
| Kuang et al., 2023 | Murine (C57BL/6) | USP2 Depletion (ML364) ± Anti-PD-1 | CD4^+^FoxP3^+^ | RM-1 (Prostate Cancer) | 5–6 mice per group | ~20 days (tumor growth monitoring) |
| Yan et al., 2015 | Preclinical mouse models (C57BL/6) | Ad-LIGHT (adenoviral vector) + PSCA TriVax (therapeutic vaccine) | CD4^+^CD25^+^FoxP3^+^ | Preclinical model (Murine prostate cancer) | Not specified | ~40 days post-tumor challenge |
| Johnson et al., 2017 | Phase I clinical trial | pTVG-HP DNA vaccine (encoding prostatic acid phosphatase) | CD4^+^CD25^+^FoxP3^+^ | Biochemically recurrent Prostate Cancer (nmHSPC) | 34 patients | Pre-treatment and post-vaccination sampling |
| Ruan et al., 2021 | Murine (C57BL/6) | Enzalutamide ± FLII overexpression (HA-FLII) | CD4^+^CD25^+^FoxP3^+^ | RM-1 (Enzalutamide resistant) | n=12 per group | 30 days (Tumor growth) / 60 days (Survival) |
| Fujita et al., 2017 | Open-label, single-arm, Phase I/II clinical trial | HVJ-E (Inactivated Sendai Virus Particles) | CD4^+^ CD25^+^ FoxP3^+^ | Phase I/II human study: CRPC patients (Docetaxel-resistant or ineligible). | 7 patients | Two 28-day treatment cycles |
| Ager et al., 2017 | Murine (C57BL/6) | STING agonist (ADU-S100) + Anti-CTLA-4 + Anti-PD-1 + Anti-4-1BB | CD4^+^ CD25^+^ FoxP3^+^ | TRAMP-C2 (Prostate Cancer) | 5 mice per group | 31 days (tumor harvest) / 100 days (survival) |
| Noguchi et al., 2016 | Randomized Phase II Clinical Trial | Personalized Peptide Vaccination (PPV) + Metronomic low-dose CPA, 50 mg/day) | CD4^+^ CD25^+^ FoxP3^+^ | mCRPC | 70 patients | 8 weekly injections (Cycle 1) followed by 8 doses at 2-3 week intervals |
| Fan et al., 2025 | Murine (C57BL/6) | Dendritic Cell-derived Extracellular Vesicles (DEX _XCL1_) + Oxaliplatin | CD4^+^ CD25^+^ FoxP3^+^ | Mouse models of CRPC (RM-1 and OVA-expressing RM-1 cells) | Not specified | ~24 days post-tumor inoculation |
| Gasparri et al., 2025 | Preclinical (mechanistic + in vivo) | 5a-HSA (peptide 5a coupled to human serum albumin) alone or with S-NGR-TNF | αvβ8^+^ (expressed by tumor-infiltrating Tregs) | TRAMP-C2 (Murine Prostate Cancer) | N/A | Varied by *in vivo* & *in vitro* experiment |
| Koga et al., 2017 | Randomized Phase II Trial | 1. PPV alone 2. Herbal medicines (Hochu-ekki-to and Keishi-bukuryo-gan) + PPV | CD4^+^CD25^+^FoxP3^+^ | mCRPC | 1. 35 patients assigned; 31 analyzed for immune response  2. 31 patients treated; 26 analyzed for immune response | 8 weekly cycles |
| Ariyan et al., 2018 | Murine (C57BL/6) | 1. Anti-CTLA-4 alone 2. Gemcitabine + CTLA-4 blockade (anti-CTLA-4) | CD4^+^FoxP3^+^ | TRAMPC2 Murine Model | Not specified | Varied (up to 60 days for survival) |
| Yuan et al., 2021 | Phase I clinical trial | Nivolumab (Anti-PD-1) + HDR Brachytherapy + EBRT + ADT | CD4^+^CD25^+^CD127^low^FoxP3^+^ | GG5 high-risk prostate cancer | 12 patients | 12 months (primary endpoint) |
| van Dodewaard‐de Jong et al., 2016 | Phase II Trial (Extension of GVAX trial) | GVAX vaccine (GM-CSF transduced cells) followed by Mitoxantrone + Prednisone | CD4^+^CD25^+^FoxP3^+^ | mCRPC (Phase I trial) | 33 patients | Median 14.3 months survival follow-up |
| Zareian et al., 2024 | Phase I clinical trial | hTERT peptide vaccine + Low-dose CPA + Celecoxib + Adjuvants (Montanide/Imiquimod) | CD4^+^ CD25^high^ CD127^low^ and FoxP3^+^ | mCRPC | 5 Pca patients | Up to 8 cycles (3-weekly); median follow-up 43.1 months |

Prostate Cancer, GG5: Grade Group 5, DNPC: Double-Negative Prostate Cancer, PSA: Prostate-Specific Antigen, ADT: Androgen Deprivation Therapy, ICB: Immune Checkpoint Blockade, EBRT: External Beam Radiation Therapy, PPV: Personalized Peptide Vaccination, GM-CSF: Granulocyte-Macrophage Colony-Stimulating Factor (an immune-stimulating protein), TRT: Targeted Radionuclide Therapy, CPA: Cyclophosphamide, FoxP3: Forkhead box P3

**Figure S1.** Risk of Bias assessment in non-randomised trials using the ROBINS-I tool.

**Figure S2.** Risk of Bias assessment in randomized clinical trials using the Cochrane RoB 2.0 tool.

**Figure S3.** Risk of Bias assessment in preclinical (animal) studies using the SYRCLE tool.
